# Mutation analysis of circulating plasma DNA to determine response to EGFR tyrosine kinase inhibitor therapy of lung adenocarcinoma patients

**DOI:** 10.1038/srep33505

**Published:** 2016-09-19

**Authors:** Anja Lisa Riediger, Steffen Dietz, Uwe Schirmer, Michael Meister, Ingrid Heinzmann-Groth, Marc Schneider, Thomas Muley, Michael Thomas, Holger Sültmann

**Affiliations:** 1 Cancer Genome Research Group, German Cancer Research Center (DKFZ) and National Center for Tumor Diseases (NCT), Im Neuenheimer Feld 460, 69120 Heidelberg, Germany; 2 Translational Lung Research Center (TLRC) Heidelberg, German Center for Lung Research (DZL), Im Neuenheimer Feld 156, Heidelberg, Germany; 3 Translational Research Unit, Thoraxklinik at University Hospital Heidelberg, Amalienstraße 5, 69126 Heidelberg, Germany; 4 Department of Oncology, Thoraxklinik at University Hospital Heidelberg, Amalienstraße 5, 69126 Heidelberg, Germany; 5 German Cancer Consortium (DKTK), Im Neuenheimer Feld 460, 69120 Heidelberg, Germany

## Abstract

Long-lasting success in lung cancer therapy using tyrosine kinase inhibitors (TKIs) is rare since the tumors develop resistance due to the occurrence of molecularly altered subclones. The aim of this study was to monitor tumors over time based on the quantity of mutant plasma DNA and to identify early indications for therapy response and tumor progression. Serial plasma samples from lung adenocarcinoma patients treated with TKIs were used to quantify *EGFR* and *KRAS* mutations in circulating DNA by digital PCR. Mutant DNA levels were compared with the courses of responses to treatment with TKIs, conventional chemotherapy, radiotherapy, or combinations thereof. Variations in plasma DNA mutation levels over time were found in 15 patients. We categorize three major courses: First, signs of therapy response are associated with a fast clearing of plasma DNA mutations within a few days. Second, periods of stable disease are accompanied by either absence of mutations or fluctuation at low levels. Finally, dramatic increase of mutational load is followed by rapid tumor progression and poor patient survival. In summary, the serial assessment of *EGFR* mutations in the plasma of NSCLC patients allows conclusions about controlled disease and tumor progression earlier than currently available methods.

Lung cancer is the leading cause of cancer related mortality worldwide^1^. Non small cell lung cancer (NSCLC) is diagnosed in 85% of the patients, most of them in a locally advanced or metastatic stage^2^, which is associated with limited therapy options and poor prognosis. In recent years, cancer genome sequencing studies have revealed numerous molecular alterations in NSCLC, which have led to the reclassification of tumor subtypes and stratified therapies. For example, the presence of mutations in the epidermal growth factor receptor (EGFR) gene qualifies NSCLC adenocarcinoma patients for targeted therapy with tyrosine kinase inhibitors (TKIs), leading to improved overall survival^3^,^4^,^5^,^6^. However, survival times are limited due to the development of TKI resistance. Thus, recognizing impending resistance with a subsequent adoption of the therapy strategy would be highly desirable. In recent years, pilot studies have demonstrated the capability to detect mutations in circulating DNA in blood plasma, reflecting the landscape and heterogeneity of primary tumors and metastases^7^,^8^,^9^,^10^,^11^. Serial evaluation of mutant plasma DNA could provide a noninvasive assessment of therapy response and tumor progression, including the detection of resistance mutations or an increase of *EGFR* sensitizing mutations associated with clinical progression^12^,^13^,^14^,^15^,^16^,^17^. Most of these studies considered assessments within long time intervals (weeks or months) after initiation of treatment^16^,^18^,^19^,^20^. Here, we describe the analysis of serial plasma DNA samples from 16 NSCLC patients under TKI therapy. We quantified prominent mutations in *EGFR* and *KRAS* genes in cell free DNA using digital PCR assays and compared these to the clinical progression data of the same patients. The aim of the study was to derive patterns of mutant plasma DNA courses over time and to evaluate the potential of this liquid biopsy approach for monitoring tumor disease and predicting therapy response.

## Results

The patient cohort comprised 16 adenocarcinoma patients under therapy at the Thoraxklinik Heidelberg from 2011 to 2016. All patients presented with stage III or IV disease and carried *EGFR* mutations as confirmed by molecular pathological analysis of tumor tissue (Table 1). One patient carried a second mutation in codon 12 of the *KRAS* gene (*KRAS* G12C). Eight patients harbored a sensitizing deletion of *EGFR* exon 19, six of the remaining eight carried an *EGFR* L858R mutation in exon 21. T790M mutation status of tumor tissue was unknown in 56.2% of the cases. The patients received TKI (erlotinib, gefitinib, or afatinib) therapy either as 1^st^-line or as treatment subsequent to surgery, chemotherapy or radiation therapy (Supplementary Table S1). At the end of the observation periods, all tumors had metastasized, and five patients had deceased with median overall survival times of nine months.

### Evaluation of DNA quantity and integrity

Plasma samples were collected between July 2014 and February 2016. The concentrations of circulating DNA in the plasma samples ranged between 7.4 and 4,768 ng/ml (median 30.7 ng/mL) and were not correlated to gender or clinical parameters such as tumor stage, therapy response, or outcome (data not shown). Size distributions of the DNA fragments varied among the samples. Frequently, DNA laddering with a prominent peak at around 166 bp could be observed (Supplementary Figure S2). Here, the smallest peaks are harboring the majority of tumor-derived DNA^21^,^22^,^23^. Variable amounts of high molecular weight DNA were frequently associated with higher cfDNA concentrations. Eight samples were excluded from further analysis due to low quality in the Bioanalyzer test.

### Determination of mutant allele fractions in plasma DNA

Following quality control, 107 plasma samples were included into the mutation analysis. Sensitizing *EGFR* mutations were detected in 61.7% (61/102) of the samples (Table 1; Supplementary Table S2). The *KRAS* mutation was present in five samples from one patient. Overall, in 15 out of 16 patients (93.7%), at least one plasma sample was positive for the known mutation. The T790M resistance mutation appeared in 17.5% (17/97) of samples, which comprised 60% (9/15) of the patients.

The digital PCR method was able to detect between 0.033 and 0.1% mutant alleles in wt background (data not shown), which is in line with previously reported sensitivities^17^,^24^. The results of the mutation analysis were compared to clinical data, including treatment plan, disease progression, and other events. In most cases, mutant allele frequencies corresponded to the tumor burden, irrespective of individual differences in therapy and disease courses. Three predominant patterns in plasma DNA levels were observed in the majority of the patients and are illustrated in the following paragraphs.

### Acute and temporary changes in cfDNA mutation levels in response to TKI therapy

**Figure 1:**
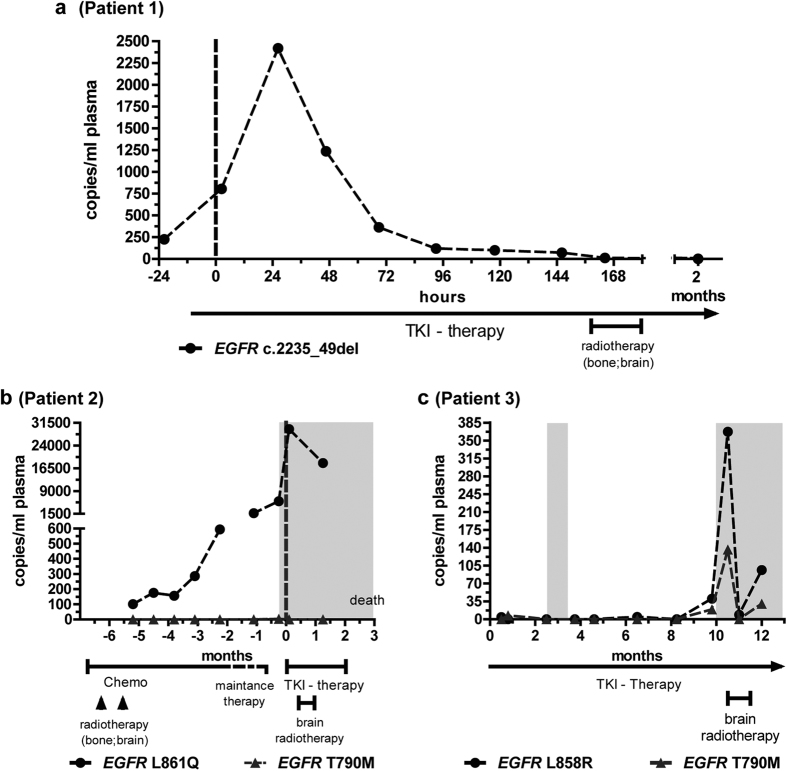
Occurrence of mutant EGFR cfDNA in response to therapy. X-axis time frames, related to the start of TKI therapy (dashed vertical lines). Information about individual treatment schedules (chemo=chemotherapy; TKI-therapy; radiotherapy) are given below the x-axis. Y-axis: numbers of mutant alleles in plasma samples (copies/ml plasma), with sensitizing EGFR mutations labeled with black dots and T790M with grey triangles. White background: oncological stable state; grey background: disease progression.

To assess the early time course of mutant cfDNA levels, changes within one week of initiation of TKI therapy were analyzed in two patients; one with daily tracking (Fig. 1A). Twenty-six hours after the first drug application (afatinib), an 11-fold increase of mutant DNA was measured, indicating DNA release from *EGFR* c.2235_49del-harboring tumor cells as a molecular response to TKI therapy^25^,^26^. The initial peak was followed by a continuous decline of mutant plasma DNA during the next two days. After 96 hours, mutation levels in plasma were lower than before therapy and further declined during the next three days. The clinical follow-up after two months was concurring with an objective therapy response and tumor remission. Concomitantly, *EGFR* sensitizing mutant alleles were no more detectable in plasma.

In the second patient (Fig. 1B), the sensitizing *EGFR* mutation had developed over the course of five months prior to TKI therapy. The patient suffered a tumor progression after 1^st^-line chemotherapy, whereupon TKI therapy was introduced. A steep rise of mutant alleles, which is consistent with the eradication of susceptible tumor cells, was observed on the fifth day after TKI therapy. However, this was in contrast to patient 1, in whom mutant alleles had dropped below the pre-therapeutic level at this time point. Radiotherapy of brain metastases in patient 2 was initiated one day after TKI onset, and a further decline of mutant alleles in plasma was observed after five weeks. However, the number of mutant alleles remained higher than prior to therapy. Tumor progression instead of therapy response was diagnosed and the patient deceased two months later.

Patient 3 had already received TKI therapy for more than 10 months before tumor progression became clinically evident with a brain metastasis, whereupon radiotherapy of the brain was initiated (Fig. 1C). One day after the first fraction, a steep increase of *EGFR* sensitizing and resistance mutations in cfDNA was observed, presumably caused by radiation-induced tumor cell death and DNA release through the blood brain barrier, which had become permeable due to radiation^27^,^28^. The identification of the T790M mutation in plasma suggested its origin from the irradiated metastasis, since T790M had not been found in the primary tumor. One day after termination of radiotherapy, cfDNA levels of the sensitizing mutation declined to almost zero, and the T790M mutation was no more detectable. MR imaging confirmed stability of the cranial metastasis. In the last sample, both mutations recurred, which was accompanied by progressive disease. These three examples suggested that plasma analysis immediately before and after therapy onset could be a valuable indicator of therapy response.

### cfDNA mutations indicating therapy response and stable disease

**Figure 2:**
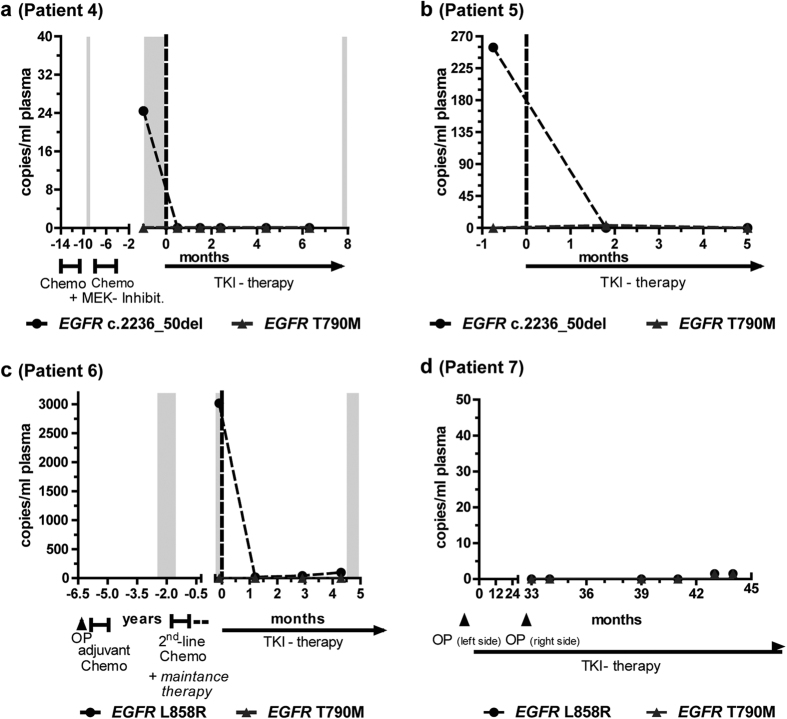
Response to TKI therapy is accompanied by a quick drop of plasma mutant DNA levels. A subsequent time interval of low mutant EGFR levels indicates a phase of stable disease. For designations, please refer to legends of Fig. 1. OP: operation/tumour resection; MEK-Inhibit.: Selumetinib therapy.

The examples illustrated above suggested that long-term response to TKI could be characterized by response immediately detectable in plasma, followed by a longer period of very low mutant cfDNA levels. These patterns could be equally highlighted in other plasma samples: Patients responding well to TKI therapy showed a strong decrease of *EGFR* mutant alleles from pre-therapeutic measurements to the next sample under therapy (Fig. 2). Interestingly, rising *EGFR* mutant cfDNA levels were observed in patient 6 two months prior to clinical progression (Fig. 2C). Overall, six (No. 1, 4, 5, 10, 11, and 16) out of seven patients with a clinical response to TKI therapy demonstrated such a complete absence of sensitizing mutations in the first assessment under EGFR TKI therapy. This was independent of prior treatments with chemotherapy or a MEK inhibitor. With continued stability of disease, *EGFR* mutant plasma levels remained low. In some cases, this trend sustained, indicating that TKI therapy could successfully be applied for even longer time periods: The monitoring of patient 7 started after 33 months of successful TKI therapy (Fig. 2D). Neither sensitizing nor T790M resistance mutations in *EGFR* could be detected in any of the six plasma samples collected over one year of TKI therapy. Patients 8 and 9 were monitored over 11 and 13 months, respectively (Supplementary Figure S3B,C). The plasma DNA mutation levels of these individuals showed slight variations on a low level, which is consistent with stable tumor disease.

### Association of *EGFR* and *KRAS* mutations with therapy failure and tumor progression

**Figure 3:**
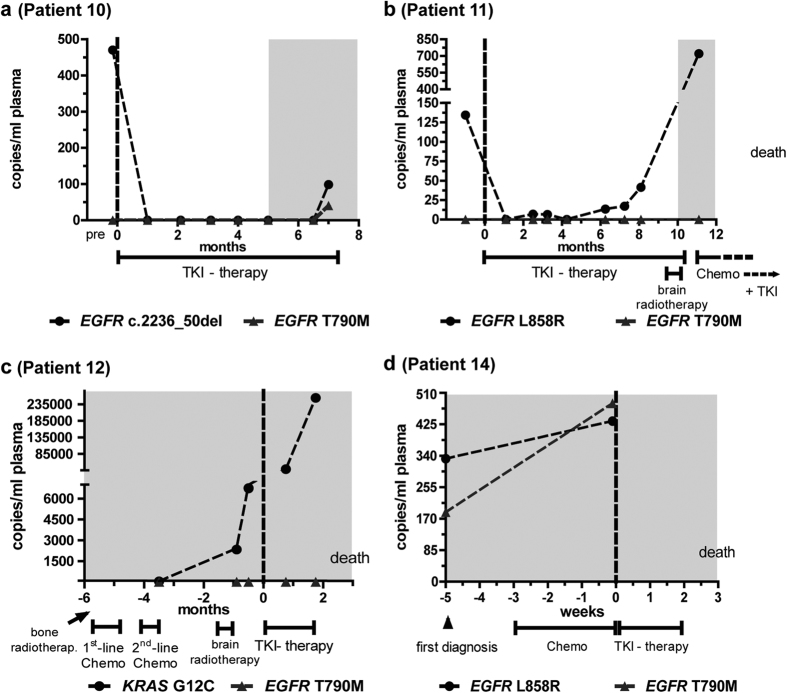
Different manifestations of tumor progression. Four different manifestations of progressive disease, all having in common that rise in plasma course reflected an objective aggravation of cancer disease. For designations, please refer to legends of Fig. 1. pre TKI: plasma sample collected prior to TKI onset.

In contrast to response and controlled tumor disease, various forms of therapy failure, such as tumor relapse after initially stable disease, progressive disease despite therapy or changes of the treatment plan, were observed in six patients (Fig. 3, Supplementary Figure S4). Tumor progression and therapy failure were associated with a detectable T790M mutation in the cfDNA of five patients (No. 3, 10, 13, 14, and 15), with variable time points of occurrence during tumor progression. In these patients, the resistance mutation exhibited a course similar to the sensitizing mutation, albeit to a lower extent and somewhat lower level. For example, in patient 10 (Fig. 3A), where T790M had not been found in tissue, tumor progression concurred with the first detection of T790M in cfDNA. Three further patients (No. 2, 3, and 13; Fig. 1B,C; Supplementary Figure S4A) exhibited detectable plasma T790M levels, although this mutation had not been detected in the primary tumor tissues and metastasis biopsies.

Patient 15 was studied during the late 1^st^-line TKI therapy with gefitinib, tumor progression, and 2^nd^ line treatment with erlotinib (Supplementary Figure S4B). The patient had a detectable T790M resistance mutation during 1^st^-line TKI treatment, which might have contributed to inefficient therapy. Initial decreases of *EGFR* sensitizing and resistance (T790M) mutations could be seen four weeks after erlotinib administration, indicating a therapeutic benefit. However, both mutations recurred only eight weeks later in the cfDNA. The disease progressed after initiation of systemic chemotherapy, and the patient deceased two months later.

T790M occurrence was associated with acquired resistance to TKI therapy but also pre-existing in TKI-naïve patients (No. 14 and 16; Fig. 3D, Supplementary Figure S3A). For example, patient 14 (Fig. 3D) exhibited high plasma levels of sensitizing and resistance *EGFR* mutations in two pre-therapeutic samples. He showed no objective response to TKI initiation, but a steady progression of tumor disease and deceased two months after the first diagnosis. No plasma samples under TKI therapy were available, but the T790M mutation had been confirmed in the biopsy of primary tumor tissue.

The courses of plasma DNA in two patients (11 and 12; Fig. 3B,C, respectively) indicated that huge increases of mutant alleles were associated with particularly poor prognosis and survival times of less than 6 months. Interestingly, in patient 11, progressive disease could already be detected in plasma several months before clinical progression. Mutant DNA levels increased 15-fold over pre-therapeutic levels within three months. Patient 12, harboring two mutations (in *EGFR* and in *KRAS*), showed no response to any therapy and died ten months after the first diagnosis. Here, disease progression was accompanied by considerably high amounts of cfDNA and a vast number of *KRAS* mutant alleles.

## Discussion

In accordance with previous reports^11^,^16^,^20^,^29^,^30^, we demonstrate that serial mutation analysis of cfDNA in plasma is feasible for therapy monitoring. Despite variances in treatment or tumor manifestation, changes in mutant cfDNA levels were detected in almost all patients, in accordance with clinical course of disease. Three major categories could be attributed: evidence for therapy response, periods of stable disease, and impending tumor progression.

While several previous studies demonstrated the feasibility of serial assessment of mutations in cfDNA, albeit at longer intervals^16^,^18^,^19^,^20^, our data suggest that it is worthwhile to examine the first time periods before and immediately subsequent to therapy onset. Therapy response based on cfDNA mutations was observed after only 26 h after first drug application and mutational load decreased in the following days, indicating that the impact of TKI therapy is largest within the first few days. This was supported by repeated plasma analysis and clinical follow-up for more than two months. In addition, the data from patient 3 show that response in plasma is not only seen during the first days after TKI application but also when radiotherapy of distant regions (brain) is applied. Unfortunately, this patient progressed only two weeks afterwards due to the deterioration of extracerebral manifestations, which was again reflected by elevated mutation levels in cfDNA. Marchetti *et al*.^13^ reported decreased plasma DNA levels on the fourth day of TKI therapy in the majority of responding patients but did not analyze the developments of cfDNA mutations in the first hours after drug application. They further noticed varying decreases in association with shorter progression free survival for some patients, defining them as *slow responders*^13^. Using instantaneous assessment of cfDNA levels after surgical resection of colorectal cancer, Diehl and colleagues could make a first prediction for therapy success^31^. Our results suggest that this strategy could be adopted for *EGFR* sensitizing mutations in TKI-treated NSCLC patients. In addition, *EGFR* T790M mutant alleles in TKI-naïve patients^20^,^32^,^33^ could provide further insights into incipient development of therapy resistance.

Our data also suggest that longer periods of controlled disease after initial therapy response are paralleled by either absence of sensitizing mutations or fluctuations at low levels. Thus, as long as this pattern is found, the mutations in cfDNA suggest sustained therapy response. However, since most of these NSCLC tumors will inevitably progress, it is important to find evidence for this ongoing process as early as possible. Our data suggest that impending tumor progression is characterized by a continuous increase of *EGFR* sensitizing (and, in several cases, resistance) mutations up to four months before clinical symptoms were observed. Thus, regular tumor monitoring using cfDNA in short time intervals would be useful to determine progression earlier than currently possible and to potentially adapt the treatment strategy accordingly. Finally, vigorous tumor progression is paralleled by the occurrence of vast numbers of mutant alleles in the plasma (*EGFR* mutations in patients 2, 11, 13, and 14, as well as, *KRAS* mutations in patient 12). This finding was associated with very short patient survival times.

Concerning minimal invasiveness of diagnostic procedures, the liquid biopsy approach is superior to the analysis of tissue biopsies, which can be hardly implemented in regular, short time intervals. Furthermore, liquid biopsies can provide extra information: For example, the frequent T790M mutation in *EGFR* had not been identified in the tissues (patients 3, 10, and 13) but was detected in the cfDNA samples. However, much more knowledge is required to corroborate the current findings before they can be translated into the clinical setting. This includes the analysis of larger and systematically controlled patient cohorts, early and more frequent sampling as well as the standardization of pre-analytical processes^34^,^35^. Furthermore, a solid understanding of the mechanisms, including spatial and temporal patterns of tumor DNA release into the bloodstream is required.

In summary, we show that early and frequent mutation analysis in plasma of advanced NSCLC patients provides molecular data, which can be correlated with clinical information. Mutations in cfDNA can be identified prior to clinically detectable progression and therefore could be used to inform earlier decisions on revised therapy strategies.

## Materials and Methods

### Patient cohort and plasma preparation

The study was approved by the local ethics committee of the Medical Faculty Heidelberg (S048/2012) with amendment (July 31, 2014). All methods were carried out in accordance with the guidelines of the Translational Lung Research (TLRC) group in the German Center for Lung Diseases. Likewise, all experimental procedures were approved by the TLRC group in the German Center for Lung Diseases. Sixteen patients with advanced or recurrent NSCLC adenocarcinoma were recruited at the Thoracic Clinic in Heidelberg. Written informed consent was obtained from all patients in accordance with the guidelines of the ethics committee of the Medical Faculty Heidelberg. Serial blood sampling was implemented over the course of treatment with either TKI or other modalities. In eleven cases, samples could be drawn prior to TKI therapy. Between two and eleven samples of peripheral blood from patients were collected in EDTA tubes (Sarstedt, Nümbrecht, Germany) and processed within one hour. Blood samples were centrifuged at 2000 × g for 10 minutes at 10 °C, plasma was withdrawn and stored at −80 °C until use.

### DNA extraction and quality control

cfDNA was extracted from 500 μL aliquots of frozen plasma with the QIAamp Blood Mini Kit (Qiagen, Hilden, Germany) according to adapted manufacturer´s recommendations (Supplementary Methods). DNA quality and fragment sizes were examined using the Bioanalyzer 2100 with High Sensitivity DNA Kit (Agilent Technologies, Santa Clara, CA, USA).

### DNA analysis using digital PCR

Absolute quantification of plasma cfDNA was performed using the Quantstudio 3D digital PCR System (Thermo Fisher Scientific, Waltham, MA, USA) and the validated *Taq*man assay for the reference gene *TERT* according to the manufacturer´s protocol (Supplementary Methods). The amount was calculated based on an external standard reference curve of fragmented genomic DNA (Roche Diagnostics, Mannheim, Germany). CfDNA from plasma samples was subjected to the measurement of known sensitizing *EGFR* and resistance (T790M), as well as *KRAS* mutations using digital PCR and TaqMan® Rare Mutation Assays (Supplementary Table S3 in supplemental methods). The chips were read using the Quantstudio 3D digital PCR instrument and the data were analyzed with QuantStudio™ 3D AnalysisSuite™ Software (example plot shown in Supplementary Figure S1). Three independent observers performed blinded analysis of the plots. Raw data for absolute quantification and rare allele detection were listed as copies/μl reaction volume. Pooled blood samples were used as controls to exclude background signals. The numbers of mutant alleles in the samples were reported as copies/mL plasma (Supplementary Methods).

## Additional Information

**How to cite this article**: Riediger, A. L. *et al*. Mutation analysis of circulating plasma DNA to determine response to EGFR tyrosine kinase inhibitor therapy of lung adenocarcinoma patients. *Sci. Rep.* **6**, 33505; doi: 10.1038/srep33505 (2016).

## Supplementary Material

Supplementary Data 1

Supplementary Table S1

Supplementary Table S2

## Figures and Tables

**Table 1:** Patient characteristics.

Patient	Gender	Age [y]	Smoking status	TNM	Tumor tissue			Plasma	
					Mutation	*EGFR* T790M	No. samples	*EGFR* sens. mut. (No. pos. samples)	*EGFR* T790M (No. pos. samples)
1	m	56	non smoker	cT4 cN0 cM1b	*EGFR* c.2235_49del	unk	10	9	n.d.
2	f	54	fmr. smoker; 35 pys	cT4 cN3 cM1b	*EGFR* L861Q	neg	9	9	1
3	m	68	fmr. smoker; 35 pys	cT2b cN2 cM1a	*EGFR* L858R	neg	11	6	4
4	f	61	cur. smoker; 20 pys	cT4 cN2 cM1b	*EGFR* c.2236_50del	unk	6	1	0
5	f	55	fmr. smoker	cT2A cN0 cM1b	*EGFR* c.2236_50del	unk	3	1	1
6	f	73	non smoker	pT1 pN1 pM0 R0	*EGFR* L858R	unk	4	4	0
7	f	57	fmr. smoker; 28 pys	pT3 pN9 pM1a R0	*EGFR* L858R	neg	6	0	0
8	f	68	non smoker	cT2A cN1 cM1b	*EGFR* L858R	unk	10	5	1
9	f	73	non smoker	cT4 cN2 cM1a	*EGFR* c.2235_49del	unk	11	6	0
10	f	60	non smoker	cT3-4 cN3 cM1b	*EGFR* c.2236_50del	neg	8	2	1
11	f	73	non smoker	cT2-3 cN2 cM1a	*EGFR* L858R	unk	9	7	0
12	m	56	cur. smoker; 25 pys	cT1a cN2 cM1b	*EGFR* c.2129 A > G; *KRAS* G12C	unk	5	5 (*KRAS*)	0
13	f	60	non smoker	pT1B pN1 pM0 R0	*EGFR* c.2236_50del	neg	3	3	3
14	m	50	fmr. smoker; 2 pys	cT4 cN3 cM1b	*EGFR* L858R	pos	2	2	2
15	f	45	non smoker	pT4 pN2 cM0 R1	*EGFR* c.2235_49del	pos	5	4	3
16	f	59	cur. smoker; 43 pys	cT4 cN3 cM1b	*EGFR* c.2235_49del	unk	5	2	1

(No.: number; y: years; sens.: sensitizing; pos: positive; m: male; f: female; fmr.: former; cur.: current; pys: packyears; unk: unknown; neg: negative; n.d. = not done).
