# Supplementary material for: Mutation analysis of circulating plasma DNA to determine response to EGFR tyrosine kinase inhibitor therapy of lung adenocarcinoma patients
Source: Sci Rep. 2016 Sep 19;6:33505. doi: 10.1038/srep33505 (PMC5027592; doi:10.1038/srep33505)
Supplement: Supplementary Data 1 [file srep33505-s1.doc]

**Mutation analysis of circulating plasma DNA to determine response to EGFR tyrosine kinase inhibitor therapy of lung adenocarcinoma patients**

Anja Lisa Riediger, Steffen Dietz, Uwe Schirmer, Michael Meister, Ingrid Heinzmann-Groth, Marc Schneider, Thomas Muley, Michael Thomas, and Holger Sültmann

**SUPPLEMENTARY DATA**

**Supplementary methods**

**DNA isolation**

Cell-free circulating DNA was isolated from 500 µL plasma using the QIAamp DNA Mini Kit (Qiagen, Hilden, Germany), with proteinase K digestion at 37°C for 1 h. In the modified protocol, 500 µL AL buffer and 450 µL of 100% ethanol were added to the samples. All centrifugation steps were carried out at 11,000xg. The remaining procedure was performed according to manufacturer´s instructions. DNA was eluted in 2x 35 µL nuclease free water and stored at -20°C until further use.

**Digital PCR**

Quantification of the total DNA and mutation analysis of all samples were carried out using the QuantStudio™ 3D Digital PCR System (Thermo Fisher Scientific, Waltham, MA, USA). The reaction solution (containing 7.25 µL Master mix, 0.71 µL TaqMan assay and 6.54 µL DNA) was loaded on the QuantStudio™ 3D Digital PCR 20K Chip. PCR was run on Thermocycler ProFlex 2x Flat PCR System (Thermo Fisher Scientific) with 96°C for 10 min followed by 44 cycles of 60°C for 2 minutes, 98°C for 30 s, followed by 2 min at 60°C. Subsequently, the signals were detected by the QuantStudio™ 3D Digital PCR Instrument. The following assays were used (Table S3):

Supplementary table S3: TaqMan Digital PCR Rare Mutation Assays

| **Assay ID** | **Assay name** | **Gene** | **Nucleotide exchange** | **Amino acid**  **Exchange** | **Wt - allele**  **(VIC labeled)** | **mutant allele**  **(FAM- labelled)** |
| --- | --- | --- | --- | --- | --- | --- |
| AHLJ0XO | EGFR_6223 | EGFR | c.2235_2249del15 | p.E746_A750delELREA | GGAATTAAGAGAAGC | - |
| AHRSROS | EGFR_6240 | EGFR | c.2369C>T | p.T790M | C | T |
| AHRSRSV | EGFR_6224 | EGFR | c.2573T>G | p.L858R | T | G |
| AH0JEUD | KRAS_516 | KRAS | c.34 G>T | p.G12C | G | T |
| AH399I0 | EGFR_6225 | EGFR | c.2236_2250del15 | p.E746_A750delELREA | GAATTAAGAGAAGCA | - |
| AHMSY3Z | EGFR_6213 | EGFR | c.2582T>A | p.L861Q | T | A |

To quantify the amounts of cfDNA, the *TaqMan® Copy Number Reference Assay, human, TERT,* was used . The amount was calculated based on an external standard reference curve of fragmented genomic DNA (Roche Diagnostics, Mannheim, Germany).

**Data Analysis**

Pre-analyzed imaging data from QuantStudio™ 3D Digital PCR Instrument was transferred to the AnalysisSuite™ Software (Thermo Fisher Scientific), and data analysis was performed following the manufacturer’s instructions. Initially, assignment of sample wells as positive or negative was dependent on their amplification status. By means of two-dimensional histograms and a synoptic dot plot diagram, the fluorescence signals were assigned to targets. Wildtype and mutated alleles were identified using VIC and FAM dyes, respectively. Empty wells without amplified templates were marked with ROX. Supplementary Figure S1 exemplarily shows a pair of histograms for VIC and FAM signals and four dot plots.

Raw data for absolute quantification and rare allele detection was reported as copies/μL with a 95% confidence interval and quality score. The total number of negative reactions was fit to a Poisson distribution to estimate the absolute copies of template molecules present in the sample volume. Mutant allele fraction was equally calculated by the Analysis Suite Software.

In the ultimate report, we listed final results as copies/mL plasma. Mutant allele number in the original plasma sample was calculated with the following equation:

Cmut_ori = (14,5µl x c_mut_ x m_ori_)/ m_V_

where Cmut_ori is the number of mutant alleles in the original sample; 14,5 µL and m_v_ are reaction volume and inserted DNA amount [ng] in the dPCR, whereas c_mut_ [copies/µL] are the measured copies; m_ori_ [ng/mL plasma] is the total cfDNA concentration. The values obtained were normalized by the use of negative controls. Background signals for mutant alleles were 0 mutant copies/mL plasma for all assays except AHRSROS (0.59 copies/mL).

**Supplementary Figure S1**


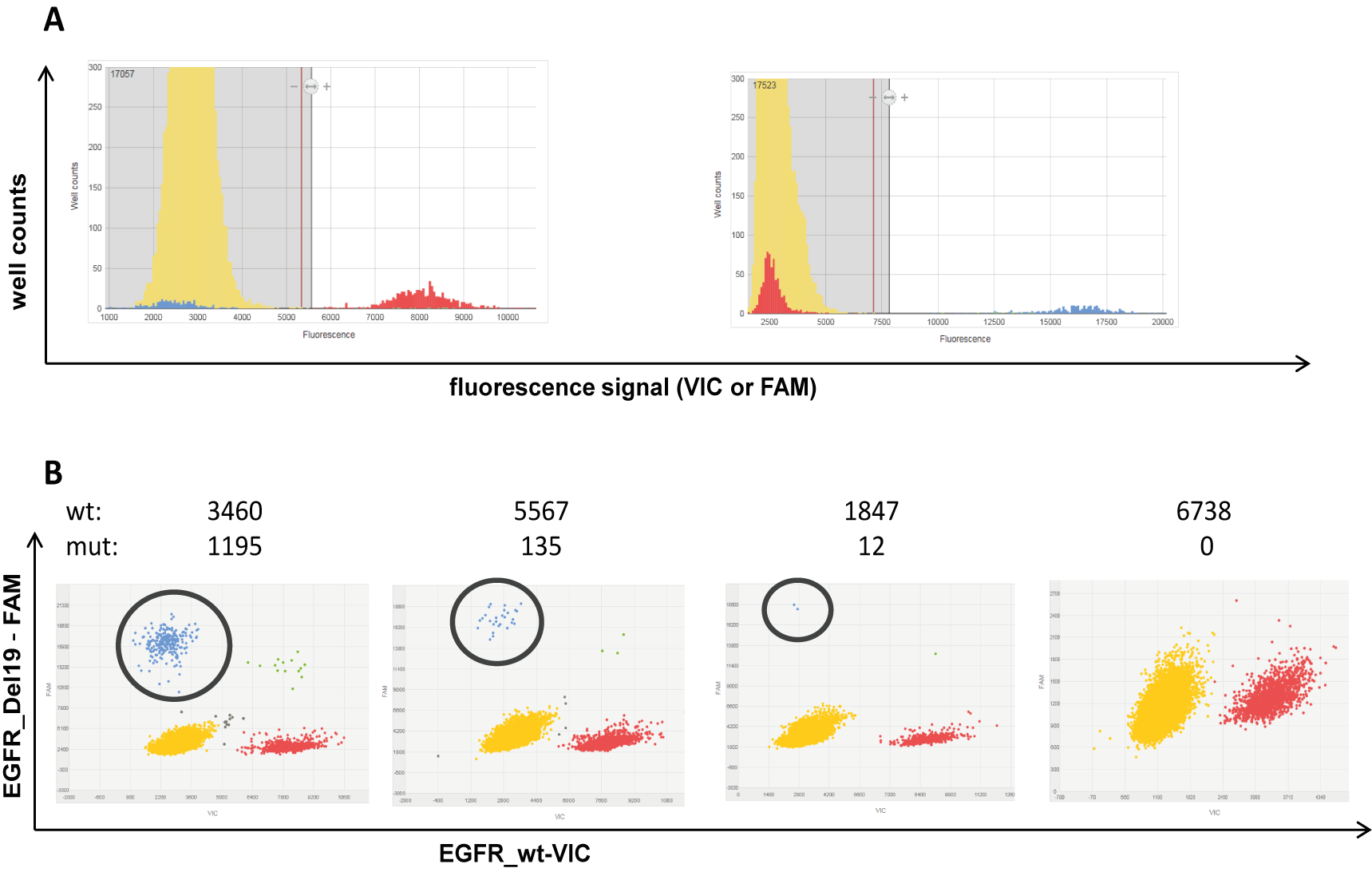


**Supplementary Figure S2**
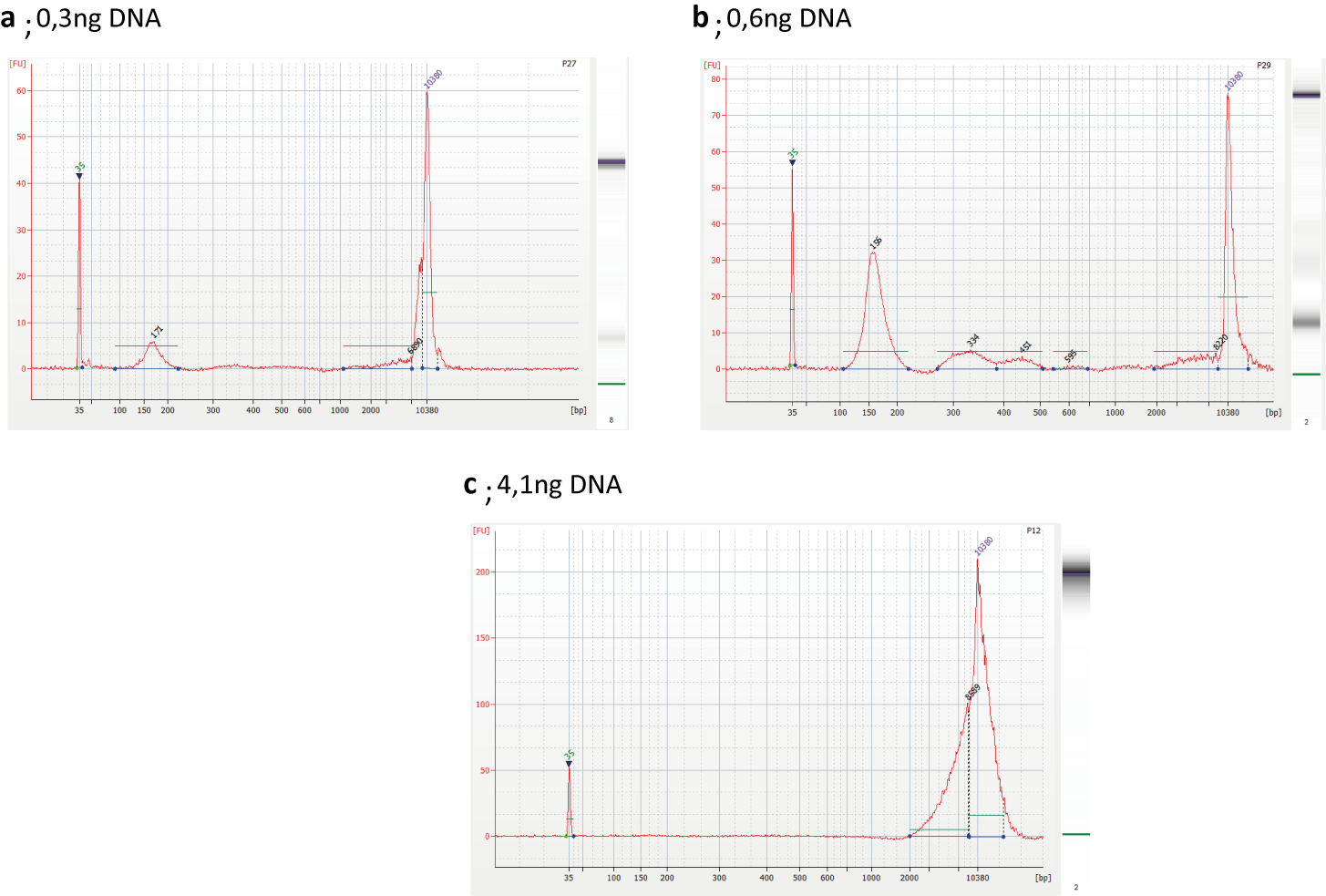


**Supplementary Figure S3
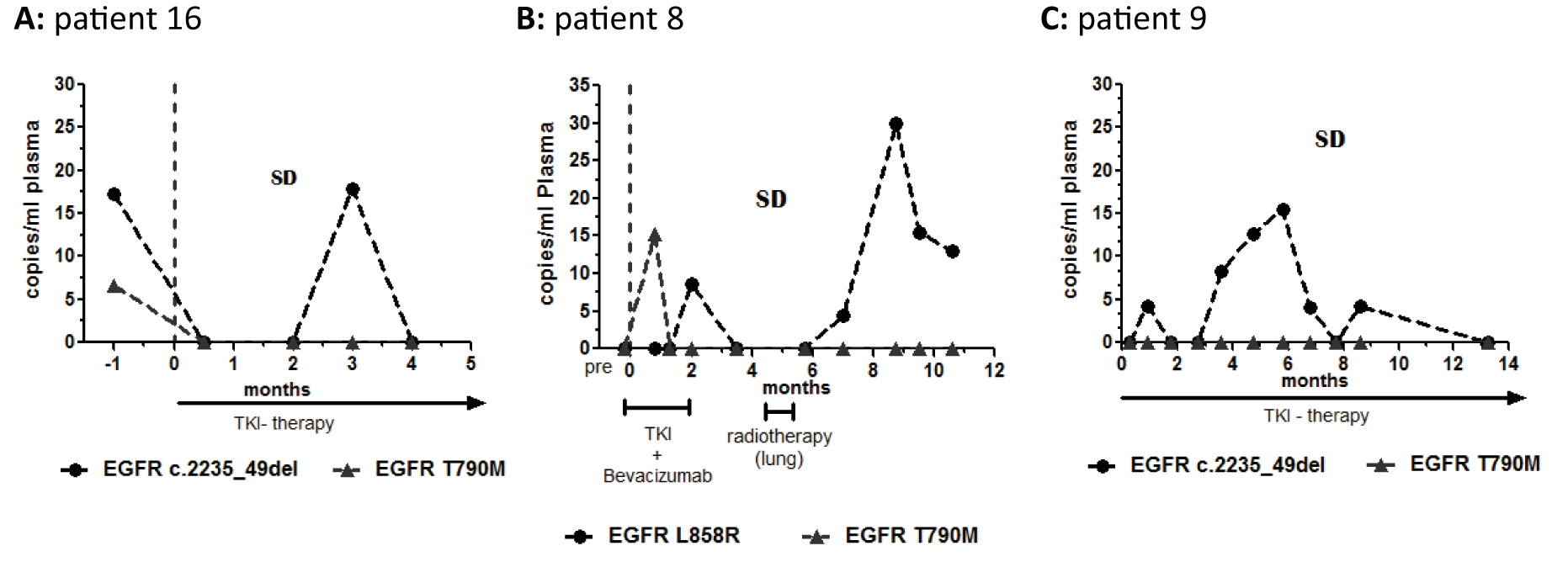
**

**Supplementary Figure S4**

**
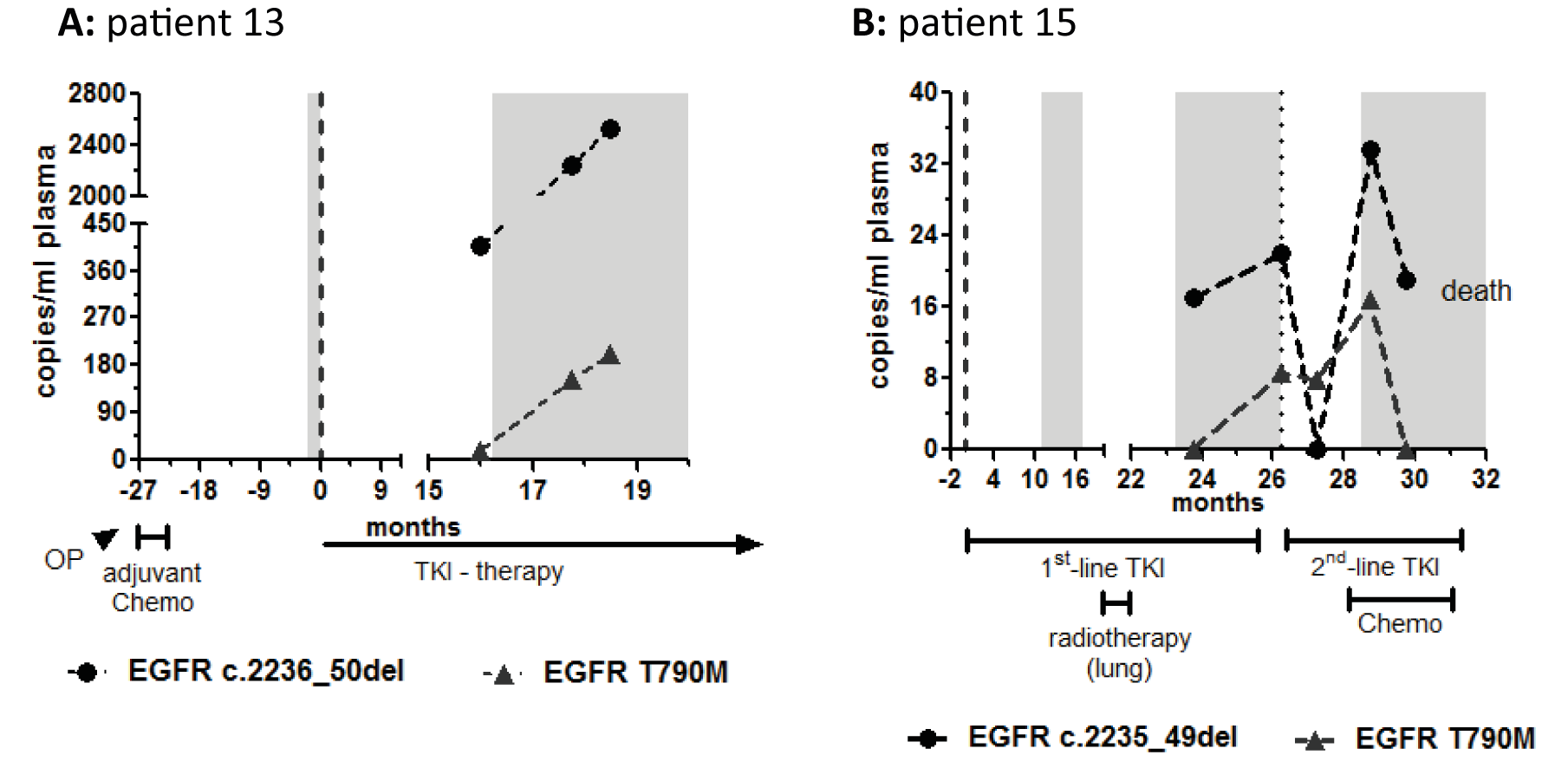
**

**Supplementary Legends**

**Supplementary figure S1.** Detection of *EGFR* sensitizing mutations. A. Quantstudio 3D Analysis Suite plots of the digital PCR raw data for the two channels, VIC (left), and FAM (right); B. Plots with processed data (red, wildtype alleles; blue, mutant alleles; yellow, no amplification signals; green, wildtype and mutant DNA present in one well). The numbers of wildtype and mutant (circled) alleles (in copies/ml plasma) are listed above the diagrams.

**Supplementary figure S2.** Integrity of circulating plasma DNA pictured with Bioanalyzer profiles. Fragments shorter than 1000 bp originate from apoptotic cells (1-3). Profile A presents the frequently observed peak at ~160 bp, ranging from 145 bp to 196 bp, which is in the range of the length of the histone- bound DNA in the nucleosome. In profile B, the prominent peak at ~160 bp is accompanied by DNA fragments two, three or four times this size (“nucleosomal laddering”). Profile C contains a high number of long DNA fragments at ~ 10 kb, probably derived from lymphocytes. High abundance of such intact genomic DNA was often accompanied by large amounts of cfDNA in the plasma samples.

**Supplementary figure S3.** Undetectable low mutant DNA levels in plasma were associated to stable disease. All three patients had an oncological stable state for up to fourteen months following the first TKI therapy. Simultaneously, mutant alleles in plasma varied on a low level. The *EGFR* T790M mutation was low in patients 16 and 8 (A and B) and not detected in patient 9 (C). The diagram features correspond to those explained in Figure 1.

**Supplementary figure S4.** Co-occurrence of increased *EGFR* sensitizing and T790M resistance mutations in plasma with disease progression. Both patients exhibited parallel developments of the *EGFR* sensitizing and T790M mutations. Mutant plasma DNA curves concurred with therapy course and disease progression. The diagram features are corresponding to the description in Figure 1. OP: operation/tumour resection.

**Supplementary table S1.** Therapy management and tissue analysis. The table lists the therapy plan for every patient by naming all treatments and substances. Additionally, available tissue samples, their origins and the EGFR mutation status, analyzed by sequencing, are reported. The collecting dates can be compared to the plasma curves (figure 1-3; supplementary figure S3-4). The numbers are related to the time intervals on the x- axis.

**Supplementary table S2.** Results of plasma mutation analysis with the digital PCR. The table specifies the measurement results of the digital PCR performance for every patient. The results are listed as wt- and mutant allele copies per ml plasma. Mutation frequencies are is given in percentages. Used Taqman assays are named according to supplementary table S3. Sample ID and collecting date, as well as cfDNA amounts of plasma samples are reported.
